# The Case for Visual Analytics of Arsenic Concentrations in Foods

**DOI:** 10.3390/ijerph7051970

**Published:** 2010-04-28

**Authors:** Matilda O. Johnson, Hari H.P. Cohly, Raphael D. Isokpehi, Omotayo R. Awofolu

**Affiliations:** 1 Department of Environmental Sciences, University of South Africa, PO Box 392, Pretoria 003, South Africa; E-Mail: awofoor@unisa.ac.za; 2 Center for Bioinformatics & Computational Biology, Department of Biology, Jackson State University, Jackson, MS 39217, USA; E-Mail: raphael.isokpehi@jsums.edu

**Keywords:** arsenic, foods, risk assessment, toxicity, visual analytics

## Abstract

Arsenic is a naturally occurring toxic metal and its presence in food could be a potential risk to the health of both humans and animals. Prolonged ingestion of arsenic contaminated water may result in manifestations of toxicity in all systems of the body. Visual Analytics is a multidisciplinary field that is defined as the science of analytical reasoning facilitated by interactive visual interfaces. The concentrations of arsenic vary in foods making it impractical and impossible to provide regulatory limit for each food. This review article presents a case for the use of visual analytics approaches to provide comparative assessment of arsenic in various foods. The topics covered include (i) metabolism of arsenic in the human body; (ii) arsenic concentrations in various foods; (ii) factors affecting arsenic uptake in plants; (ii) introduction to visual analytics; and (iv) benefits of visual analytics for comparative assessment of arsenic concentration in foods. Visual analytics can provide an information superstructure of arsenic in various foods to permit insightful comparative risk assessment of the diverse and continually expanding data on arsenic in food groups in the context of country of study or origin, year of study, method of analysis and arsenic species.

## 1. Introduction

Arsenic is a naturally occurring toxic metal and its presence in food could be a potential risk to the health of both humans and animals [1]. Inorganic arsenic occurs naturally in soil, air and water a well as through anthropogenic sources such as mining, agriculture and non-agricultural activities [2]. Arsenic in contaminated soils may cause adverse effects due to inhalation of dust and ingestion of contaminated soils. Arsenic toxicity is a global health problem affecting millions of people. Arsenic-contaminated groundwater is often used for food and animal consumption, irrigation of soils, which could potentially lead to arsenic entering the human food chain [1].

Prolonged ingestion of arsenic contaminated water may result in manifestations of toxicity in all systems of the body. Disease caused by this long-term exposure to arsenic include pigmentation, hyperkeratosis, many other cardiovascular, neurological, hematological, renal and respiratory diseases, as well as cancer of the skin, lung, bladder, liver, kidney and prostate [3]. The most serious concern is the potential of arsenic as a carcinogen [4]. Symptoms typically do not appear for two to ten years from the start of chronic exposure, and they may also appear long after exposure ceases [5].

Visual Analytics is a multidisciplinary field that is defined as the science of analytical reasoning facilitated by interactive visual interfaces [6,7]. Furthermore, visual analytics combines techniques from computer-based information visualization with techniques from computational transformation and analysis of data. The concentrations of arsenic vary in foods making it impractical and impossible to provide regulatory limit for each food. Furthermore, the risks of naturally occurring arsenic in foods have received less attention when compared to drinking water and airborne workplace exposure [8]. However, exposure to total and inorganic arsenic from diet is significantly higher than from drinking water [9]. Uneyama *et al.* [10] have collated data on arsenic content in six food groups (crops, milk/meat/egg, fish, algae, seafood and others) to provide a comprehensive comparison data that may be helpful to risk managers and consumers. This review article presents a case for the use of visual analytics approaches to provide comparative assessment of arsenic in various foods. This article is divided into (i) metabolism of arsenic in the human body; (ii) arsenic concentrations in various foods; (ii) factors affecting arsenic uptake in plants; (ii) introduction to visual analytics; and (iv) benefits of visual analytics for comparative assessment of arsenic concentration in foods.

## 2. Metabolism of Arsenic in the Human Body

The small intestine is the site of absorption of arsenic through an electrogenic process involving a proton gradient with an optimal pH of 5 [4]. The metabolism of inorganic arsenic in the human body is one of the crucial determinants of the toxicity resulting from exposure to inorganic arsenic [11]. The metabolic conversion of inorganic arsenic to methylated products is through a multistep process that results in mono-, di-, and trimethylated arsenicals [12,13]. Arsenate (pentavalent arsenic) is reduced to arsenite (trivalent arsenic) which is the preferred substrate for methylation which is an oxidative process [13,14].

The liver is the site for methylation of inorganic arsenite (iAs^III^) through a folate dependent one-carbon metabolism [11,15]. The entry of arsenic into hepatocytes is controlled by membrane transporters including water transport proteins aquaporin [11]. Folate contributes the methyl groups used in the generation of s-adenosylmethionine (SAM). Arsenic methyltransferase (AS3MT) transfers the methyl group from SAM to inorganic arsenite (iAs^III^) to generate monomethylarsonic acid (MMA^v^). After MMA^v^ is reduced to monomethylarsonous acid (MMA^III^), AS3MT can catalyze a second methylation to generate dimethylarinic acid (DMA^v^). Inorganic arsenic and its methylated metabolites are mostly excreted in urine in 4–5 days so there is a decreased rate of bioaccumulation [16]. Methylation was thought to be a detoxification process of inorganic arsenic but is increasingly recognized as a pathway of inorganic arsenic activation because the methylated forms are more cytotoxic, more genotoxic, and more potent inhibitors of the activities of some enzymes than the inorganic containing arsenic in the trivalent oxidation state [13,17,18].

In chronic arsenic ingestion, arsenic accumulates in the liver, kidneys, heart, lungs with smaller amounts in the muscles, nervous system, gastrointestinal tract and spleen [19]. The toxicity features to humans of water soluble inorganic arsenic are presented in Table 1. It is now well recognized that people in Bangladesh are exposed to arsenic mainly through the food ingestion, which is through the consumption of contaminated drinking water and large amounts of rice and other foods (vegetables, dal, fish, milk, chicken and other meats) [20].

## 3. Arsenic Concentrations in Various Foods

The data on arsenic in six food groups have been collated by Uneyama *et al.* [10]. In this section, the emphasis is to highlight published articles that have compared arsenic content of selected foods obtained from arsenic-endemic regions of Bangladesh and West Bengal India with other parts of the world. Furthermore, comparison data on arsenic concentrations in parts of vegetables and grains as well as those found in algae and seafood from Spain and USA respectively are presented.

In Bangledesh, irrigation with underground water has led to increase in the arsenic content of surface soils which then increases the arsenic content of irrigated crops including rice (*Oryza sativa*) and could negatively impact crop yield [21]. In an investigation of arsenic content in soils of two agroecological regions of Bangledesh, the concentration of inorganic arsenic in non-calcareous soil (pH around 6.5, free calcium carbonate absent) was 4.85 to 12.20 mg/kg while that of calcareous soil (pH around 7.6, free calcium carbonate present) was 11.60 to 24.40 mg/kg [22]. Furthermore, in soil samples around arsenic-enriched Singair areas of Bangledesh, the inorganic arsenic content ranged from 40 to 60 mg/kg [22]. In a investigation of rice grain samples from 214 households in 25 arsenic-endemic Bangladeshi villages, the Rahman *et al.* [23] found that total arsenic content ranged from 2 μg/kg to 557 μg/kg dry weight (dw). The arsenic concentrations in control samples obtained from South Australia ranged from 3 μg/kg to 87 μg/kg dw, significantly lower (*p <* 0.001) than those collected in the contaminated areas.

Al Rmalli *et al.* [1] in a survey of arsenic in foodstuffs on sale in the United Kingdom and imported from Bangladesh, found the concentration of total arsenic in vegetables from Bangladesh ranged from 5 to 540 μg/kg, with a mean of 54.5 μg/kg. Furthermore, the concentration of total arsenic in freshwater fish ranged between 97 and 1318 μg/kg, with a mean value of 350 μg/kg. In the case of freshwater fish, Puti (*Puntius gonionotus*) had a very high arsenic concentration of 1,318 μg/kg with a mean of 580 μg/kg in its dried forms. The total arsenic concentrations of some selected vegetables including carrots, radish, potatoes, broccoli and cabbage grown in the United Kingdom (UK)/European Union (EU) showed the mean and range of arsenic concentrations to be 24.2 and 5 to 87 μg/kg respectively. The highest concentrations were 87.2 μg/kg for marrow and 68.5 μg/kg for cabbage. The comparison of the UK/EU vegetables versus the vegetables imported from Bangladesh, the mean arsenic concentrations are approximately 2- to 3-fold higher for the latter.

Roychowdhurg *et al.* [24] surveyed total arsenic content in food collected in Jalangi and Domkal blocks from the arsenic-affected area of West Bengal, India. The food categories surveyed were vegetables (92 and 123 μg/kg), cereals and baked goods (156 and 294 μg/kg) and spices (92 and 201 μg/kg) (mean arsenic concentrations for Jalangi and Domkal blocks respectively).

Arsenic concentrations in anatomical parts of vegetables and crops increase in the following order; grain << leaf < stem <<< root. Studies on rice [25,26], beans [27,28] observed elevated concentrations of arsenic in plant roots compared to other plant tissue. Analysis of arsenic concentrations in chard, radish, lettuce and mung beans showed that arsenic accumulated in the following order: root >>> shoot > leaf [2]. Furthermore, speciation studies demonstrated that root, shoot and leaf tissue contained only inorganic arsenic with no organic arsenic species identified.

Most arsenic in seafood is organic which is less toxic than inorganic species [8]. In Valencia (Spain), the highest levels of total arsenic in algae food products was obtained from brown algae, followed by red algae with green algae having the lowest concentrations [29]. Burger and Gochfield [30] found in a study of heavy metals in commercial fish in New Jersey, USA that some of the fish in the study (Chilean sea bass, croaker, flounder, porgie, and whiting) had arsenic levels of over 1.3 ppm regulatory limit by the United States Environmental Protection Agency (EPA).

## 4. Factors Affecting Arsenic Uptake in Plants

Four geochemical mechanisms of natural arsenic pollution are reductive dissolution, alkali desorption, sulphide oxidation, and geothermal activity [31]. Furthermore, many soil factors influence the amount of arsenic available for plant uptake including include redox potential, pH, the contents of organic matter, iron, manganese, phosphorus and calcium carbonate, and soil microbes [32]. The influence of some of these soil properties and constituents also varies significantly within the year in soils that alternate between anaerobic and aerated conditions, as occurs in seasonally-flooded soils and irrigated upland soils used for paddy cultivation.

Plant uptake of arsenic from soils is complicated by a number of factors. In aerated soils used for crops such as wheat, maize and most vegetables, arsenic is present mainly as As(V) and as such is likely to be in the solid phase. Therefore, in such soils, arsenic in groundwater used for irrigation is quickly absorbed by iron hydroxides and becomes largely unavailable to plants. In anaerobic soil conditions such as occur in flooded paddy fields, arsenic is mainly present as As(III) and is absolved in the soil-pore water (the soil solution) [33]. It is the more readily available to plant roots.

## 5. Visual Analytics

### 5.1. Goal of Visual Analytics

Analysis is both an art and a science. The goal of analysis is to make judgments about an issue or larger questions. The focus areas of visual analytics are summarized in Table 2. The perception is that visual analytic techniques are developed for massive datasets and complex problems. Chabot [34] argues that visual analytics techniques are for everyday use for both large and small multidimensional data as well as for answering simple and complex questions. In addition, it is not always about finding hidden insights about the data, but exploring, cleaning, gaining confidence in, summarizing, pursing inconclusive paths, confirming facts and presenting findings about the data. In other words, visual analytics is an iterative process that involves collecting information, data preprocessing, knowledge representation, interaction, and decision making [35]. In summary, the goal of visual analytics tools is to enable people apply computing operations to data by interacting directly with visual representations. There are state of the art research on visualizing and analyzing environmental and public health from geospatial aspects including cancer [36–38] and zoonotic (animal to human) diseases [39–41]. The objective of this section is to introduce visual analytics and demonstrate the potential benefits for visualizing and analyzing large data on arsenic in foods.

### 5.2. “Insight” in Visual Analytics

“Insight” in visual analytics has quite a few definitions but none is commonly accepted as a definition by the community of visualization [42–44]. Researchers in the area of cognitive neuroscience define insight as that ‘eureka’ moment, when a person moves from the point of not knowing the solution to a problem to the point of knowing. It is detectable by measuring the neural activity using an Electroencephalography (EEG) or functional Magnetic Resonance Imaging (fMRI) [45]. This is a spontaneous moment [46] and often the thought process leading up to this solution occurred in a subconscious state [47]. The community of visualization defines insight as “the gaining of knowledge about a data after interactively visualizing and exploring it”. It is thus knowledge building and not spontaneous. They also define insight as “new information discovered that could bring to light previously unknown relationships in the data” [48]. To measure the amount of knowledge building insight, the methods used to gather the knowledge are evaluated as well as studies to measure the amount of knowledge gained by a user. Thus, in visual analytics and information visualization, insight can be discovered, gained or provided whereas in cognitive science, insight is experienced making it an event and now a substance. It has been proposed that spontaneous insight in fact comes from knowledge about a problem and each spontaneous insight can open up new directions for more knowledge building.

### 5.3. Visual Analytics as an Integrated Approach

Analytically important data are buried in vast streams of all types. Raw data, are rarely appropriate for direct analysis hence visual analytics must bring all relevant data into a single consistent analytical context, regardless of the form in which the information began, to support analysis and discovery [49].

Computer-based information visualization centers on helping people to explore or explain abstract data through interactive software that exploits the capabilities of the human perceptual system (InfoVis 1997—IEEE Symposium on Information Visualization). Information visualization draws on the intellectual history of several traditions, including computer graphics, human-computer interaction, cognitive psychology, semantics, graphic design, statistical graphics, cartography, and art.

**Figure 1.**
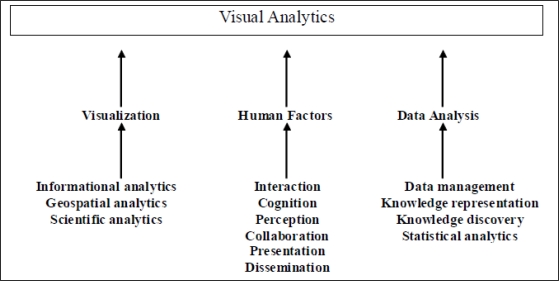
Visual Analytics as an integrated approach.

Visual analytics is more than visualization but is an interdisciplinary field of research with a scope involving many fields including knowledge discovery, information analytics amongst others. It draws strength from these other fields in order to gain insight into data of various sizes and complexity. It is also an integrated approach combining fields such as visualization, human factors and data analysis which in turn integrates different methodologies as shown in Figure 1.

### 5.4. Challenges in Visual Analytics

There are several application and technical challenges in visual analytics [35]. Three aspects that are common to the challenges are described below.

**Information overload**: The improved ability to collect and store data is currently growing faster than the ability to analyze it [35]. There are often infinite possibilities in terms of mappings and views and there is a high potential for information overload in dense information fields. The amounts of data to be visualized currently exceed the pixels on the display and thus needs to be reduced using data reducing techniques such as aggregation, filtering, compression.

**Visual scalability**: The capability of visualization tools to effectively display large datasets in terms of either the number or the dimension of individual data elements [50]. Scalability is a challenge of visual analytics because it determines the ability to process large heterogeneous datasets (such as those genomic datasets) by means of computational overhead as well as appropriate rendering techniques.

**Interpretability**: The ability to recognize or understand the data. Sometimes raw data comes with a lot of quality problems including outliers, missing values, double counts and incomplete data. The challenge is to provide a visual analytic application that will be able to clean up the data or make the analyst aware of the shortcomings of the data.

## 6. Benefits of Visual Analytics for Comparative Assessment of Arsenic Concentration in Foods

Arsenic has been shown to be a very toxic element, particularly inorganic arsenic. It has been recognized as a human carcinogen and intake must be limited [4,46]. Arsenic toxicity is a global problem affecting millions of people particularly in Bangladesh and West Bengal, India [1,4,23]. The concentrations of arsenic vary in different foods; also it is not always possible to distinguish the form of arsenic in a food. This makes it impractical, almost impossible to provide regulatory limit for each food.

Several regulatory bodies worldwide including the Joint FAO/WHO Expert Committee on Food Additive, Food Standards Australia New Zealand, World Health Organization and United States Environmental Protection Agency have set various guideline levels for total arsenic, inorganic arsenic and organic arsenic levels in various foods and drinking water. These regulatory levels are expected to help consumers, risk managers, policy makers and responsible authorities minimize exposure of humans and animals to this toxic element.

The collated data on arsenic in foods by Uneyama *et al.* [10] aims to provide a comprehensive comparison data that may be helpful to risk managers and consumers. The data is provided mostly in the form of tables. Some studies have shown that users are able to receive more information, see relationships in data more easily, save time and ultimately make more rational decisions when data is presented using visual analytic tools particularly an interactive tool as opposed to tabular representations [51–53]. Additional comprehensive data on arsenic in foods can also be obtained from the United States National Health and Nutrition Examination Survey from 1991 to 2005 [54].

**Figure 2.**
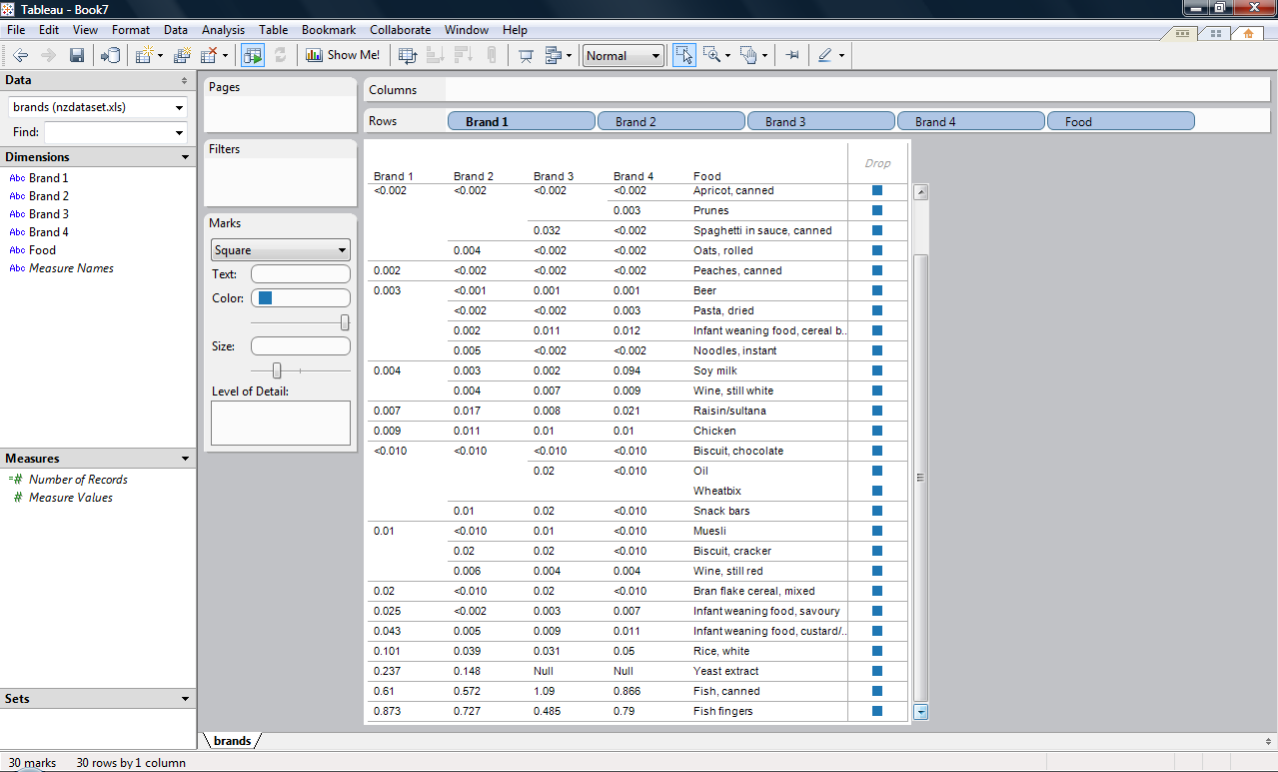
Screenshot of visual analytics interface for grouping arsenic content (mg/kg) of foods from a study in New Zealand ^a^. Visual Analytics process revealed relationship between Oil and Wheatbix. ^a^ http://www.nzfsa.govt.nz/science/research-projects/total-diet-survey/reports/quarter-2/quarter-2-nztds.pdf.

We demonstrate these benefits using selected data from analytics results of the 2003/04 New Zealand Total Diet Survey (NZTDS) [55]. The tabulated data in Table 3 was processed using Tableau [34], a visual analytics software, to visualize groupings of foods according to total arsenic content (mg/kg) (Figure 2).

**Figure 3.**
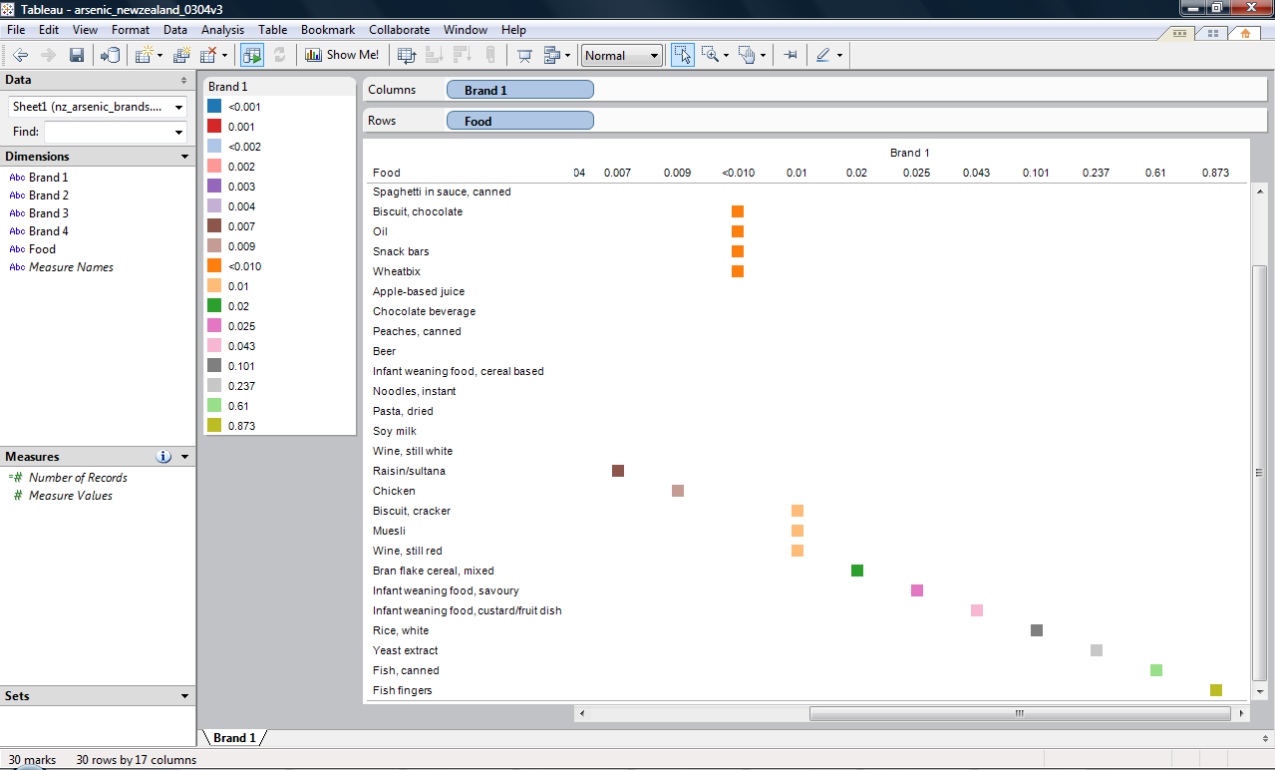
Screenshot of visual analytics of arsenic content (mg/kg) of food from a study in New Zealand ^a^. ^a^ http://www.nzfsa.govt.nz/science/research-projects/total-diet-survey/reports/quarter-2/quarter-2-nztds.pdf.

The process included connecting the spreadsheet file containing Table 3 into Tableau and subsequently the Dimensions (Brand 1, Brand 2, Brand 3, Brand 4, and Food) were dragged to the Rows panel. The analysis on the tabulated data was performed using the Text Tab (Cross-tab) feature from the Show Me! option (Analysis Menu Option). The visual analytics software automatically grouped the dataset and enabled manual sorting of the arsenic concentration. The analytics process revealed that fish foods had the highest content of total arsenic. In addition, among other relationships was that Oil and Wheatbix had the same levels of arsenic for the four brands. This observation was not initially obvious from the data. The user of the software was able to select a brand and visualize the groupings of the foods (Figure 3). This user interaction with the data can be done with any other brand.

In conclusion, visual analytics can provide an information superstructure of arsenic in various foods to permit insightful comparative risk assessment of the diverse and continually expanding data on arsenic in food groups in the context of country of study or origin, year of study, method of analysis and arsenic species.

## Figures and Tables

**Table 1.** Selected toxicity features of water soluble inorganic arsenic compounds a.

**Feature**	**Description**
Absorption	Gastrointestinal tract and lungs
Distribution	Liver, kidney, lung, spleen, aorta, and skin
Excretion	Urine at rates as high as 80%
Symptoms of Acute Inorganic Arsenic	Nausea, anorexia, vomiting, epigastric and abdominal pain, and diarrhea.
Poisoning in human	Dermatitis (exfoliative erythroderma), muscle cramps, cardiac abnormalities, hepatotoxicity, bone marrow suppression and hematologic abnormalities (anemia), vascular lesions, and peripheral neuropathy (motor dysfunction, paresthesia).
Effect of Severe Exposures	Acute encephalopathy, congestive heart failure, stupor, convulsions, paralysis, coma, and death.
General symptoms of chronic arsenic poisoning in human	Weakness, general debility and lassitude, loss of appetite and energy, loss of hair, hoarseness of voice, loss of weight, and mental disorders.
Primary target organs	Skin (hyperpigmentation and hyperkeratosis), nervous system (peripheral neuropathy), and vascular system.
Other symptoms of chronic arsenic poisoning in human	Anemia, cancer, leukopenia, hepatomegaly, and portal hypertension.

a Adapted from The Risk Assessment Information System: http://rais.ornl.gov/tox/profiles/Arsenic_ragsa.shtml.

**Table 2.** Visual Analytics Focus Area Techniques a.

**Focus Area**	**Function to Users**
Analytical reasoning	Obtain deep insights into the data at hand that will directly support assessment, planning and decision making
Visual representations and interaction	See, explore, and understand large amounts of information at once
Data representations and transformations	Convert data which may previously have appeared in all types of conflicting and dynamic into ways that support its visualization and analysis
Support production, presentation and dissemination of results of analysis	Communicate the information in the appropriate context to a variety of audience

a Adapted from [7].

**Table 3.** Total arsenic content (mg/kg) of selected foods from a study in New Zealand a.

**Food**	**Brand 1**	**Brand 2**	**Brand 3**	**Brand 4**
Apple-based juice	0.001	< 0.001	0.002	0.003
Apricot, canned	< 0.002	< 0.002	< 0.002	< 0.002
Beer	0.003	< 0.001	0.001	0.001
Biscuit, chocolate	< 0.010	< 0.010	< 0.010	< 0.010
Biscuit, cracker	0.010	0.020	0.020	< 0.010
Bran flake cereal, mixed	0.020	< 0.010	0.020	< 0.010
Caffeinated beverage	< 0.001	< 0.001	< 0.001	< 0.001
Chicken	0.009	0.011	0.010	0.010
Chocolate beverage	0.001	< 0.001	< 0.001	< 0.001
Fish fingers	0.873	0.727	0.485	0.790
Fish, canned	0.610	0.572	1.090	0.866
Infant weaning food, cereal based	0.003	0.002	0.011	0.012
Infant weaning food, custard/fruit dish	0.043	0.005	0.009	0.011
Infant weaning food, savoury	0.025	< 0.002	0.003	0.007
Muesli	0.010	< 0.010	0.010	< 0.010
Noodles, instant	0.003	0.005	< 0.002	< 0.002
Oats, rolled	< 0.002	0.004	< 0.002	< 0.002
Oil	< 0.010	< 0.010	0.020	< 0.010
Pasta, dried	0.003	< 0.002	< 0.002	0.003
Peaches, canned	0.002	< 0.002	< 0.002	< 0.002
Prunes	< 0.002	< 0.002	< 0.002	0.003
Raisin/sultana	0.007	0.017	0.008	0.021
Rice, white	0.101	0.039	0.031	0.050
Snack bars	< 0.010	0.010	0.020	< 0.010
Soy milk	0.004	0.003	0.002	0.094
Spaghetti in sauce, canned	< 0.002	< 0.002	0.032	< 0.002
Wheatbix	< 0.010	< 0.010	0.020	< 0.010
Wine, still red	0.010	0.006	0.004	0.004
Wine, still white	0.004	0.004	0.007	0.009
Yeast extract	0.237	0.148		

a http://www.nzfsa.govt.nz/science/research-projects/total-diet-survey/reports/quarter-2/quarter-2-nztds.pdf.
